# Sulforaphane-Activated Functional Nucleic Acids for Cancer Therapy: Mechanisms, Delivery Strategies, and Nanomedicine Advances

**DOI:** 10.3390/ijms27094033

**Published:** 2026-04-30

**Authors:** Mukesh Kumar, Nasir A. Ibrahim, Shafiq Ur Rahman, Kevaun Altamon George Wilson, Salwa Eman, Nosiba S. Basher, Walid Elfalleh, Mohamed Osman Abdalrahem Essa, Ahmed A. Saleh, Hosameldeen Mohamed Husien, Mengzhi Wang, Xiaodong Guo

**Affiliations:** 1 College of Animal Science and Technology, Yangzhou University, Yangzhou 225009, China; dh24021@stu.yzu.edu.cn (M.K.); kevaun.wilson@case.edu.jm (K.A.G.W.); mh24126@stu.yzu.edu.cn (S.E.); elemlak1339@yzu.edu.cn (A.A.S.); 008643@yzu.edu.cn (H.M.H.); mzwang@yzu.edu.cn (M.W.); 2 Department of Biology, College of Science, Imam Mohammad Ibn Saud Islamic University (IMSIU), Riyadh 11623, Saudi Arabia; nsbasher@imamu.edu.sa (N.S.B.); wbelfallah@imamu.edu.sa (W.E.); 3 College of Veterinary Medicine, Yangzhou University, Yangzhou 225009, China; mh24102@stu.yzu.edu.cn (S.U.R.); dh23054@stu.yzu.edu.cn (M.O.A.E.); 4 College of Veterinary Medicine, Albutana University, Rufaa 22217, Sudan; 5 Animal and Fish Production Department, Faculty of Agriculture (Al-Shatby), Alexandria University, Alexandria City 11865, Egypt; 6 Joint International Research Laboratory of Agriculture & Agri-Product Safety of MOE, Yangzhou University, Yangzhou 225009, China

**Keywords:** cancer therapy, Functional Nucleic Acids (FNAs), targeted drug delivery, Sulforaphane (SFN), SFN-based therapies, chemoprevention

## Abstract

Cancer therapy is increasingly shaped by the need for agents that are both mechanistically precise and clinically tolerable. Sulforaphane (SFN), a dietary isothiocyanate enriched in cabbage-family vegetables such as cauliflower and Brussels sprouts, has emerged as a pleiotropic modulator of tumor biology. This review synthesizes current evidence that SFN regulates diverse cancer-relevant processes, including redox homeostasis, cell-cycle progression, apoptosis, autophagy and epigenetic remodeling, largely through coordinated effects on transcriptional (for example, Nrf2, MAPK, NF-κB and AP-1), post-transcriptional (microRNAs and messenger RNAs) and epigenetic (DNA methyltransferases and histone deacetylases) networks. We then examine how functional nucleic acids, including aptamers, small interfering RNAs, microRNAs and tetrahedral *DNA* nanostructures, can be engineered to guide SFN to tumor cells, amplify pathway-specific effects and overcome resistance. Particular emphasis is placed on nanotechnology-enabled delivery platforms that enhance SFN stability, bioavailability and tumor selectivity. Finally, we outline key challenges, such as context-dependent Nrf2 activity, inter-individual variability in metabolism and incomplete clinical validation, and propose priorities for translating SFN-based functional nucleic acid systems into rational, combination-ready strategies for precision oncology.

## 1. Introduction

Globally, cancer has emerged as a leading and deadly illness among people. The World Health Organization reported that cancer was responsible for the death of over 10 million people in 2020 [1]. Cancer remains one of the leading causes of death worldwide. There are more than 200 cancer types, and researchers still lack a comprehensive understanding of their origins, tumorigenesis, and disease progression [2]. Cancer is characterized by the uncontrolled proliferation of abnormal cells, leading to metastasis and affecting various tissues and organs [3]. Risk factors contributing to cancer include physical, chemical, and biological carcinogens and lifestyle choices such as tobacco use, unhealthy diet, and physical inactivity. Disparities in cancer incidence and mortality rates exist across regions, influenced by access to healthcare, awareness, and early detection capabilities [4]. The specific delivery of newly developed anticancer drugs to tumor sites while maintaining high effectiveness is paramount because such drugs provide enhanced benefits and fewer adverse reactions. Healthcare professionals use chemotherapy alongside radiotherapy as the main treatment for cancer.

Health recommendations for high-risk cancer patients will likely benefit from chemopreventive compounds, as these compounds increase survival potential through basic food choices available at retail stores [5]. Research indicates that consuming cruciferous vegetables can decrease the total cancer risk, including colon, lung, and prostate cancer, mainly during the initial stages of the disease. The sulforaphane (SFN) compound can stop chemically induced cancers in animal studies and suppress tumor growth [6]. Talalay and Zhang were the first to isolate SFN from broccoli. SFN is an aliphatic isothiocyanate whose precursor is glucoraphanin, found in cruciferous vegetables such as broccoli sprouts, cauliflower, cabbage, and Brussels sprouts, which are 20–50 times more effective in chemoprevention than mature heads [7]. SFN is a small-molecule compound that exhibits anti-inflammatory properties and protects against oxidative damage [8].

**Figure 1:**
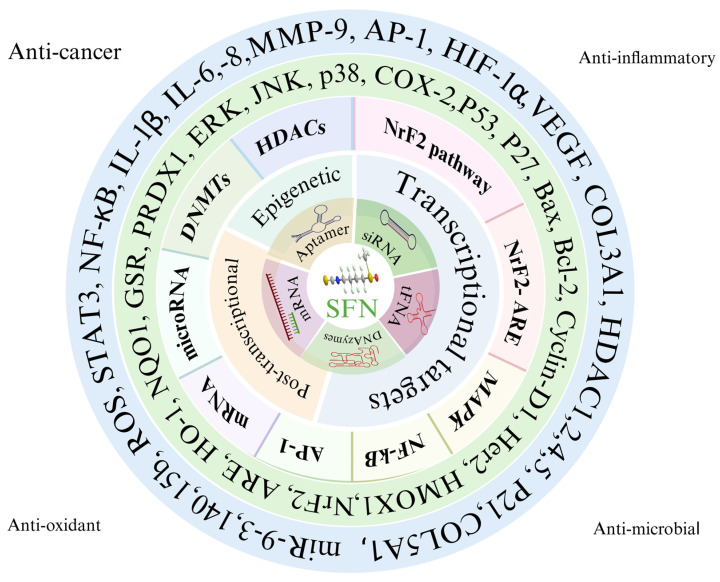
Multi-target molecular mechanisms of SFN. The circular schematic illustrates the pleiotropic effects and key molecular targets/pathways modulated by the compound, categorized into major functional sectors: anti-cancer, anti-inflammatory, anti-oxidant, anti-microbial, epigenetic, and transcriptional regulation. Central elements represent core mechanisms (e.g., DNA structure for epigenetic modifications, transcription symbols for gene regulation, and ARE/Nrf2-related icons). Outer and inner rings highlight interconnected targets and pathways, including NF-κB, Nrf2, HDACs, AP-1, STAT3, MAPK family members (ERK, JNK, p38), HIF-1α, VEGF, COX-2, IL-6, IL-1β, p53, Bax, Bcl-2, cyclin D1, p21, miRNAs, mRNA regulation, DNMTs, PRDX, GSR, HO-1, NQO1, and others. Solid and overlapping sectors emphasize crosstalk among antioxidant response (e.g., Nrf2-ARE pathway), inflammation suppression (e.g., NF-κB inhibition), epigenetic modulation (HDACs, DNMTs), and cell cycle/apoptosis regulation, underlying the compound’s broad therapeutic potential in cancer prevention, inflammation-related diseases, oxidative stress, and microbial defense.

Research shows that anticancer properties of SFN involve the regulation of biological pathways and genes responsible for triggering programmed cell death, cell cycle arrest, and inhibition of angiogenesis. SFN activates nuclear factor erythroid-2-related factor *2* (Nrf2), resulting in the upregulation of multiple cytoprotective genes that mediate cellular stress responses to oxidative stress. Research has proven that SFN acts as a cancer growth inhibitor by modifying several pathways that contribute to cancer development (Figure 1). Many scientific studies reveal that SFN functions as a dietary supplement, enhancing the efficiency of standard chemotherapeutic drugs by lowering their unwanted side effects [9,10]. It showcases tumor-reducing properties through apoptosis and cell cycle arrest by activating caspase and cellular events in the G2/M phase, which protects against colorectal cancer [11].

Genes that control proliferation, differentiation, and apoptosis are transformed by environmental pressures involving abnormal cellular changes during carcinogenesis [12]. Cancer development is characterized by multiple genetic and epigenetic changes that ultimately result in unregulated cellular proliferation and malignancy. Cancer development is regulated by essential epigenetic changes and microRNAs that control cellular gene expression [13]. Cancer contains billions of cells originating from a specific primary cell, which reproduces clonally before evading apoptosis to acquire genetic (and/or epigenetic) alterations that lead to the formation of a neoplastic cell [14]. The suppression of apoptosis in cells subjected to substantial genetic (or DNA) damage permits the survival and progressive clonal expansion of genomically unstable cells, thereby constituting a critical mechanism in the initiation and multistep progression of malignancy. Resistance mechanisms complicate survival after chemotherapy and radiotherapy because cancer cells use DNA repair pathways, cell cycle dysregulation, and cancer stem cells to protect themselves during interventions. Studies on these mechanisms have guided the development of targeted treatments and improved treatment methods, as they provide direction for diagnostic indicators and therapeutic approaches that combat resistance.

SFN is a chemopreventive and anticancer agent that controls essential cellular pathways while presenting targeted delivery options through functional nucleic acids. This review hypothesizes that SFN may improve the performance of standard chemotherapeutic drugs through key signaling pathways involved in cancer development. The targeted delivery of SFN using FNAs decreases the toxicities associated with conventional anticancer medicines and unwanted side effects of traditional cancer therapies. Additional therapeutic benefits can arise when SFN is used in combination with chemopreventive agents and functional nucleic acids, which boost its anticancer effects [15]. In addition, this review systematically explores the regulatory roles of SFN in key molecular targets associated with carcinogenesis, encompassing transcriptional, post-transcriptional, functional nucleic acid, and epigenetic pathways. Future studies should focus on improving SFN delivery methods and studying its combination effects with other therapeutic medicines before conducting clinical trials to demonstrate its safety and treatment results for cancer. Therefore, we reviewed the research progress on SFN’s complex molecular mechanisms of SFN in various cancer types. A complex understanding of these systems will establish new possibilities for cancer therapy. This review establishes pathways for utilizing SFN in cancer prevention and treatment.

## 2. SFN: A Natural Anticancer Compound

### 2.1. Chemical Properties, Sources of SFN, and Its Biological Effects

**Figure 2:**
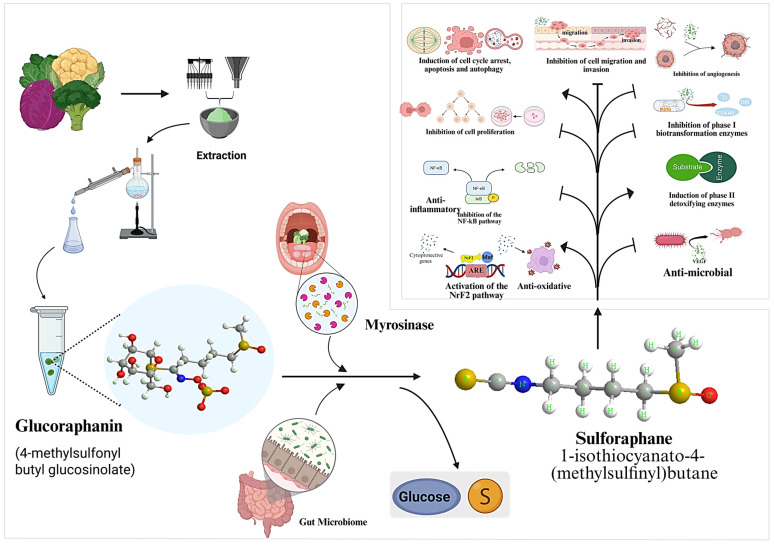
Schematic overview of the extraction, bioconversion, and molecular mechanisms of action of SFN derived from cruciferous vegetables. Glucoraphanin, the precursor glucosinolate present in vegetables such as broccoli and cauliflower, undergoes extraction followed by myrosinase-mediated hydrolysis in the presence of the gut microbiome (or plant myrosinase) to yield SFN. SFN exerts multiple chemopreventive and therapeutic effects, including activation of the Nrf2 pathway (leading to antioxidant and phase II detoxification enzyme induction via ARE/Maf binding), inhibition of NF-κB signaling and phase I metabolizing enzymes, induction of cell cycle arrest, apoptosis, and autophagy, suppression of cell proliferation, migration, invasion, angiogenesis, and metastasis, as well as antimicrobial activity. Chemical structures of glucoraphanin and SFN are shown, with key functional groups highlighted (e.g., glucose moiety in glucoraphanin and isothiocyanate group in SFN). Arrows indicate major pathways and biotransformation steps (Generated with App.biorender.com).

SFN (1-isothiocyanate-4-(methylsulfinyl) butane), a naturally occurring isothiocyanate (ITC) compound, is present in the highest quantities in broccoli sprouts among cruciferous vegetables such as cabbage, cauliflower, and kale [16]. Broccoli sprouts contain optical isomers (R and S) of the asymmetric sulfur atom in this compound. The liquid form of SFN appears as a faint yellow color, has a molecular weight of 177.28 g/mol, and contains the molecular structure C_6_H_11_NOS_2_. The cryothermal property of SFN was measured as 74.6 °C, although it exists in the range of 58.6 °C to 91.2 °C. SFN dissolves in DMSO and methanol, as well as in water-soluble substances [16,17]. The side chain components contain one of three chemical group types: aliphatic, aromatic, or heterocyclic, which establish the physical and biological properties of the isothiocyanates molecules [18]. Extraction is typically performed using aqueous and organic solvents such as methanol, ethanol, and acetonitrile [19], followed by purification via solid-phase extraction, liquid–liquid extraction, and high-performance liquid chromatography (HPLC) [20]. The content of SFN varies significantly depending on plant source, with broccoli sprouts containing substantially higher levels of glucoraphanin (20–50 mg/g dry weight) compared to mature broccoli (0.1–1.0 mg/g fresh weight) [21]. Following enzymatic conversion, SFN yields can reach approximately 1–10 mg/g dry weight in sprouts, making them one of the richest natural sources. However, the final SFN yield is strongly influenced by myrosinase activity, processing conditions, and storage stability. SFN synthesis begins when plant cell walls are broken down by chewing or cutting actions under the influence of the myrosinase enzyme, either originating from plant material components or gut microbial populations [22], which, along with inactive Epithiospecifier proteins (*ESP*), produce SFN, Figure 2. When myrosinase operates together with activated *ESP*, the reaction produces SFN nitrile [23]. Cellular entry of sulforaphane results in glutathione (GSH) conjugation, which subsequently undergoes metabolism via the mercapturic acid pathway [22]. Sulforaphane accumulation is more likely to occur in cancer cells because of their elevated glutathione content, which strengthens the anticancer properties of SFN [24].

**Figure 3:**
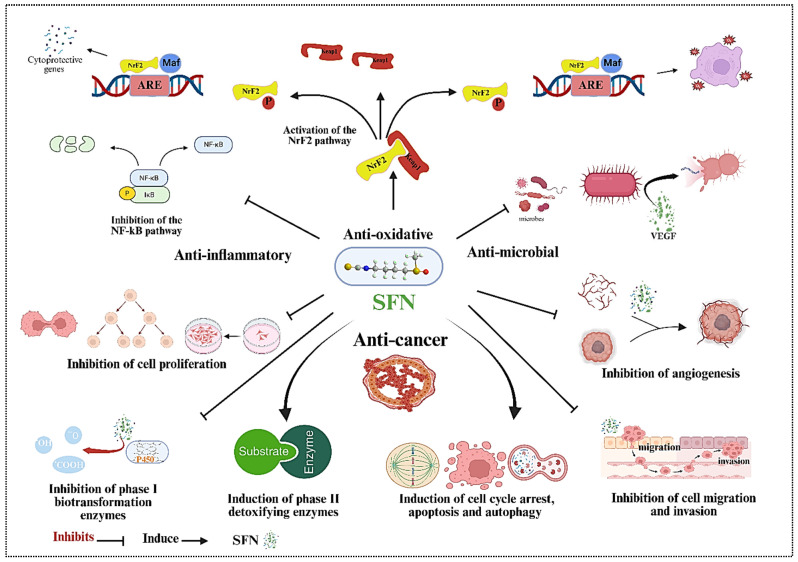
Schematic representation of the multi-targeted mechanisms of action of SFN, a bioactive isothiocyanate derived from cruciferous vegetables. The central SFN molecule (depicted as a chain-like structure) orchestrates pleiotropic effects across four major protective categories: anti-oxidative, anti-inflammatory, anti-microbial, and anti-cancer (Generated with App.biorender.com).

Many scientific investigations have documented SFN’s health advantages since its discovery. This biologically active phytochemical possesses antioxidative, antimicrobial, cytoprotective, cardioprotective, anticancer, anti-inflammatory, and other biological properties (Figure 3). Studies have shown that SFN is a potent immune system enhancer and toxin-cleansing agent [25,26]. The central beneficial aspect of SFN is its ability to fight cancer. Scientists have established that SFN fights cancer through cell proliferation repression, cellular cycle arrest, and apoptosis stimulation. Research findings indicate that SFN demonstrates anticancer effectiveness and chemopreventive actions in breast, ovarian, prostate, colon, gastric, and lung cancers [27,28].

SFN has been shown to inhibit phase I carcinogen-activating enzymes (cytochrome P450, CYP). It induces phase II detoxification enzymes [glutathione S-transferase (GST), N-acetyltransferase (NAT), and sulfotransferase (SULT)], triggering cell cycle arrest, apoptosis, and autophagy, while inhibiting angiogenesis, invasion, and metastasis [29]. Cytotoxic compounds, known as ITCs, function exceptionally well to initiate the Nrf2-Keap1-ARE signaling cascade, establishing their role in regulating cellular redox reaction dynamics, Figure 3. The Nrf2 induction mechanism is a primary defense mechanism that protects cells from free radical damage and mutagen-induced oxidative stress, preventing cellular damage [30]. Activated Nrf2 in cancer cells promotes survival mechanisms, enhances proliferation, and reduces apoptosis to create cancer-resistant characteristics [31,32].

Research indicates that SFN and other naturally occurring isothiocyanates activate Nrf2-dependent pathways to exert antioxidant effects [33,34]. SFN activates the trophic genome of Nrf2 to produce an operational state in the cells. Animal organs possess Nrf2, a transcription factor necessary for fighting oxidative stress caused by endogenous and exogenous chemicals. Through Nrf2 activation, the body controls gene expression with cytoprotective properties by regulating protective proteins that produce antioxidant, anti-inflammatory, anti-glycation, and other defensive agents [35].

### 2.2. Anti-Carcinogenic Mechanisms of SFN

SFN demonstrates antiproliferative effects on tumor cells by inhibiting cell cycle progression, inducing apoptosis, and inhibiting Histone deacetylase (HDAC) activity. Irregular expression of cell cycle proteins generates aberrant cell multiplication, resulting in tumor formation [36]. Three key checkpoints manage cell cycle progression: the G1/S checkpoint (restriction point), G2/M DNA damage checkpoint, and spindle assembly checkpoint. Therefore, Cancer treatment requires targeting cell cycle checkpoints, as disrupting the cell cycle is an essential therapeutic approach. Ongoing research showed that SFN induces significant G2/M cell cycle arrest in BC cells, preventing their proliferation [37]. It has been demonstrated that SFN increase *TP53* and *CDKN1A* expression (encoding P53 and P21 proteins, respectively) in gastric cancer cells, inducing apoptosis by inhibiting the S-phase cycle [27,38].

Apoptosis is a vital regulatory death cycle that maintains internal homeostasis by removing unwanted, damaged, and infected cells. The majority of available chemotherapy treatments cause cancer cell death by triggering apoptosis [39,40]. Glioblastoma cell apoptosis occurs when SFN treatment elevates cleaved Caspase-3 and *Bax* concentrations while lowering *Bcl-2* levels and generating ROS, leading to cell death through apoptosis and suppresses cancer development by blocking cancer cell STAT3 signaling activation [41]. HDAC is involved in various cancers because it suppresses transcription and disrupts cell cycle and apoptotic regulatory mechanisms. SFN, an active agent, inhibits HDAC enzymatic activity in cancer cells [42]. The mechanism of SFN involves reducing DNA methyl transfer (DNMT) function, particularly *DNMT1* and *DNMT3B*, to protect against cancer development through cell apoptosis and cycle arrest [43,44].

Isothiocyanates, such as SFN, inhibit angiogenesis and metastasis during the progression stage. Vascular endothelial growth factor (VEGF) is the leading cytokinin that initiates angiogenesis through its ability to boost vascular permeability while encouraging the activity of proteolytic enzymes. Fibroblast growth factor 2 (FGF-2), VEGF, and epidermal growth factor (EGF) play essential roles in tumor angiogenesis, which enables insufficient nutrient and oxygen supply [32]. The Hypoxia-inducible factor-1α (HIF-1α)-activated signaling pathway advances cell mobility and accelerates metastasis. Research has shown that SFN affects angiogenesis in colon (HCT116) and gastric cancer (AGS) cell lines. Studies have proven that SFN prevents HIF-1α expression in both HCT116 as well as AGS cancer cell lines but diminishes VEGF production in HCT116 cells [45]. Through metastasis, tumor cells migrate and invade to create new areas of disease spread, enabling them to detach from the original tumors and relocate throughout the body. The complete metastatic process requires the activation of proteolytic enzymes, such as matrix metalloproteinases (MMPs). High levels of MMPs are present in cells that develop into cancer. The results showed that SFN decreased cell invasion by repressing AP-1 and NF-κB signaling pathways triggered by ROS and reduced *MMP-9* expression caused by nicotine in gastric cancer cells [46]. SFN blocks HIF-1, VEGF, and *MMPs-2* as well as *MMPs-9* while inhibiting metastatic and angiogenic pathways in HT-29 colon cancer cells [47,48], Table 1.

The cellular process of autophagy enables the degradation of proteins, lipids, and organelles. Cells require autophagy as a basic component and fundamental metabolic process to conduct self-repair operations and maintain internal stability. Autophagy in cancer provides metabolic substances to established tumors, further contributing to their growth [49]. In vitro and in vivo studies have established that SFN treatment reduces *CD44v6* and *YAP1* expression, along with their downstream genes and EMT markers, and inhibits squamous carcinoma cell growth, NOD Scid Gamma (NSG) mouse characteristics, and their properties, while suppressing tumor sphere formation, cellular invasion, and movement [50,51].

**Table 1:** Mechanisms of SFN in Cancer Therapy.

Mechanism	Description	Key Targets	Cancer Types	References
**Apoptosis Induction**	SFN triggers programmed cell death via caspase activation and mitochondrial dysfunction.	Caspase-3, Caspase-9, *Bcl-2*, *Bax*, p53, p21	Breast, Prostate, Colon,	[52]
		Caspase-3, *Bax*, ROS, *Bcl-2*, STAT3	Glioblastoma (Malignant Brain Tumor)	[41]
		*Bax*, p53, p21	Lung, Pancreatic	[28]
**Cell Cycle Arrest**	SFN induces cell cycle arrest at G2/M or G1/S phases, preventing cancer cell proliferation by modulating cyclins and CDKs.	Cyclin B1 (*CCNB1*), *CDK1*, *CDK5R1*,	Gastric, Osteosarcoma,	[37]
		p21, p53, *CDK1*,	Bladder cancer	[53]
		p21, p53	Gastric,	[38]
**Epigenetic Modulation**	SFN inhibits HDACs and DNMTs, leading to the reactivation of tumor suppressor genes.	*HDAC1*, *HDAC2*, *HDAC6*	Prostate, Breast,	[42]
		*DNMT1*, *DNMT3B*	Colon, Lung,	[43]
		RT4, J82	Breast cancer cell	[54]
**Antioxidant Response**	SFN activates the Nrf2/ARE pathway, enhancing detoxification and antioxidant enzymes.	Nrf2, Keap1, *NQO1*, HO-1(*HMOX1*), *GSR*	Colon, Lung, Breast cancer	[55]
		HO-1, *NQO1*, *GSR*,	Bladder cancer	[53]
**Inhibition of Angiogenesis and Metastasis**	SFN inhibits angiogenesis by blocking VEGF and HIF-1α and reduces metastasis by inhibiting MMPs.	VEGF, HIF-1α,	Colon, Breast	[32]
		*MMP-2*, *MMP-9*	Lung, Gastric	[46]
		253J, EJ, 5637, T24, J82	Breast cancer cells	[56]
**miRNA Regulation**	SFN modulates miRNA expression, affecting oncogene and tumor suppressor activity.	*miR-21*, *miR-29b-3p*, *miR-199a-5p*	Colorectal, Breast cancer,	[51]
		*miR-21*, *miR-199a-5p*	Skin Squamous Cell Carcinoma	[57]
**Nrf2 Pathway Activation**	SFN activated NrF2 to enhance antioxidant response	*NQO1*, HO-1, *GST*	bladder cancer, Colon,	[53]
		*GSR*, *PRDX1*	Lung, Pancreatic	[58]
**NF-κB Pathway Inhibition**	SFN inhibits NF-κB, reducing inflammation and cancer cell survival.	IL-6, IL-8, NF-κB, *NLRP12*	Pancreatic, Breast cancer,	[59]
		NF-κB, IL-6, IL-8	Colon cancer	[60]

### 2.3. Bioavailability and Pharmacokinetics of SFN

Phytochemicals are molecules found in various plants that are used in medical science for disease management through traditional and mainstream practices. The decision to use phytochemicals, including SFN, as therapeutic agents depend on a thorough evaluation of their metabolic patterns, pharmacokinetic behavior, and bioavailability. The body shows strong absorption of SFN, with reported absorption efficiency reaching up to ~80% under conditions where active myrosinase is present, owing to its lipophilic profile and chemical composition [61]. The body quickly absorbs SFN until it reaches maximum plasma concentrations within two hours after consumption [62]. Glutathione S-transferase helps accelerate the GSH-giving reactive electrophilic carbon activity from the isothiocyanate functional group (–N=C–S). SFN is rapidly absorbed but quickly metabolized and excreted, limiting its retention, although it can temporarily accumulate within cells [63]. Absorption and pharmacokinetics data are consistent, showing that SFN is rapidly absorbed and largely eliminated from plasma within 24 h post-dosing in both human and animal studies. Repeated intake can produce tissue storage in animal models [64].

Pharmacokinetic analysis showed that patients who took 200 μmol SFN in capsule form with SFN-rich powder achieved serum levels of 0.7 ± 0.2 μM at 3 h, while the elimination half-life for SFN equivalents was 1.9 ± 0.4 h based on the mass spectrometry results [65]. In a study, Sprague Dawley adult female rats were exposed to 150 μmol SFN by mouth, plasma concentrations of SFN-derived dithiocarbamates reached peak levels (Cmax) at approximately 1 h post-ingestion, with an elimination half-life of 6–7 h [66]. SFN bioavailability depends on GRN precursor, the presence of myrosinase enzyme, and physiological conditions that negatively affect absorption rates when stomach acidity reduction drugs, such as omeprazole, impact myrosinase function. The hydrolysis of glucosinolates inside the human body depends on the presence of gut microbiota, which breaks down these compounds and boosts SFN absorption [67,68]. SFN availability also depends on individual metabolic variations, processing, and storage stability, as these factors determine its actual biological availability [69].

## 3. FNAs for Cancer Therapy

These are short, single-stranded synthetic nucleic acid sequences that perform selected biological functions outside the genetic functionalities of DNA and RNA. Therapeutic focus in FNAs continues to increase because of their multiple applications in biotechnology, medicine, and diagnostics. Numerous researchers have described FNAs, such as aptamers, DNAzymes, and other nucleic acid equivalents, due to their high binding affinities combined with chemical stability as valuable molecular tools [51]. FNAs function differently from structural nucleic acids (DNA and RNA), maintaining nucleic acid complexes, and catalytic nucleic acids, which perform biochemical reactions. Scientists have used FNAs for target recognition and reaction acceleration, which proved their importance in therapeutics and diagnostics [70]. FNAs can control immune function and enable medication transport into cells and cancer cells. These functions make FNAs powerful biomedical and biotechnological tools in therapeutics and diagnostics.

**Figure 4:**
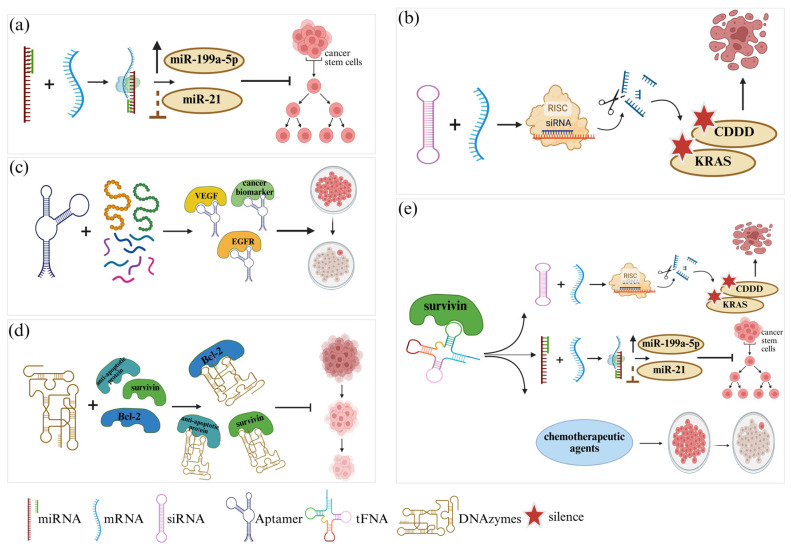
Schematic representation of nucleic acid-based therapeutic strategies for targeting cancer stem cells and overcoming chemoresistance. (**a**) Modulation of miRNA expression using antisense oligonucleotides or mimics. The addition of *miR-199a-5p* mimic inhibits cancer stem cell properties, while inhibition of *miR-21* (via antisense) reduces stemness. (**b**) RNA interference using siRNA or DNAzymes delivered via carriers (e.g., nanoparticles). siRNA is processed by the RNA-induced silencing complex (RISC) to silence target genes such as ***KRAS***, while DNAzymes (marked by ★) catalyze cleavage of complementary target mRNAs. (**c**) Targeting receptor tyrosine kinases or related pathways. Aptamer or other nucleic acid ligands bind to EGFR (or similar targets like VEGF), leading to receptor downregulation or inhibition in cancer cells. (**d**) Combinatorial targeting of anti-apoptotic pathways. Aptamers or tRNA-derived fragments (tRFNA) bind to Bcl-2 family proteins, while other nucleic acids silence surviving (*BIRC5*) and *miR-21*, promoting apoptosis in cancer cells. (**e**) Integrated approach combining miRNA modulation and gene silencing to sensitize cancer stem cells to chemotherapeutic agents. *miR-199a-5p* upregulation and *miR-21* downregulation (via inhibitors or mimics) synergize with siRNA-mediated silencing of *BIRC5* and *KRAS* (★ indicates silencing effects), reducing tumor stem cell populations and enhancing response to chemotherapy (Generated with App.biorender.com).

All FNAs that belong to the nucleic acid family, such as DNA and RNA, exhibit biological activities, including target binding and reaction acceleration abilities. The primary types of FNAs are small interfering RNAs (siRNAs), microRNAs (miRNAs), and aptamers, Table 2 and Figure 4.

Small interfering RNA (siRNA) mediates gene silencing through RNA interference (RNAi) by cleaving target messenger RNA (mRNA) molecules in the cytosol to stop gene translation [71], Figure 4. siRNAs have significant clinical value because they enable the treatment of multiple diseases, including cancer, viral infections, and genetic disorders. Research involving siRNA has demonstrated pancreatic cancer cell *KRAS* gene knockout, which leads to decreased cell multiplication without harmful side effects [72]. MicroRNAs (miRNAs) regulate cell proliferation, cell death, apoptosis, metabolic functions, and immune responses. Improper activation of miRNAs leads to numerous human diseases, including cancer, cardiovascular diseases, diabetes, neurodegenerative disorders, and autoimmune diseases [73]. Research on miRNAs as therapeutic agents has increased because these molecules regulate gene expression post-transcription. The regulatory effects of miRNAs in cancer include their oncogenic and tumor-suppressor activities, which affect cell growth processes, apoptosis, and metastasis, among other essential pathways [74]. Research has demonstrated that miRNAs serve as important regulatory factors with great potential to improve disease detection and therapeutic development across multiple medical conditions [75].

Aptamers are synthetic single-stranded DNA or RNA oligonucleotides that have gained extensive interest because they display strong target affinity in concert with high specificity for different targets, such as proteins, peptides, small compounds, and entire cells [76]. Systematic Evolution of Ligands by Exponential Enrichment (SELEX) selection process to identify target-specific aptamers with high binding capabilities [77]. Short sequences of 20–100 bases RNA or ssDNA develop aptamer structures that allow them to identify specific molecules through targeted interactions with antibody-level selectivity and affinity. Aptamers have better features than antibodies because of their quick and reasonable manufacturing process [78,79]. The aptamer can bind various target molecules, including nucleic acids, proteins, sugars, phospholipids, and all cells. These molecules have extensive functional potentials because they can be used in diagnostic examinations, sensing capabilities, therapeutic applications, and environmental detection systems [80], (see Figure 4). The therapeutic usage of aptamers has reached its peak in cancer treatment, as they function to exactly detect tumor cells while restricting unwanted side effects for improved gene therapy efficacy. Furthermore, FNAs also consist of ribozymes and deoxyribozymes that represent nucleic acid enzymes that demonstrate biochemical catalytic abilities [81]. FNA tools have versatile applications in scientific research because they are used for biosensing and therapeutic creation.

**Table 2:** FNAs in Cancer Therapy.

FNA Type	Description	Mechanism	Applications in Cancer Therapy	Delivery Systems	References
**siRNA**	Small interfering RNA that induces gene silencing through RNA interference.	Gene silencing by degrading target mRNA, reducing oncogene expression.	Silencing *KRAS*, *CD44*, and other oncogenes; enhancing apoptosis in pancreatic and breast cancer.	Lipid nanoparticles, viral vectors, polymeric nanoparticles	[71,82,83]
**miRNA**	Small non-coding RNA molecules that regulate gene expression post-transcriptionally.	Binding to target mRNA, inhibiting translation or promoting degradation, modulating tumor suppressors.	Upregulation of *miR-199a-5p*, downregulation of *miR-21*; inhibition of cancer stem cell activity.	Exosomes, lipid-based carriers, gold nanoparticles	[73,84]
**Aptamers**	Synthetic single-stranded DNA or RNA oligonucleotides with high target specificity.	High-affinity binding to target proteins, peptides, and cells, delivering therapeutic agents.	Targeting VEGF, EGFR, and other cancer biomarkers; enhancing drug delivery specificity.	Aptamer-nanoparticle conjugates, viral-like particles	[78,85]
**DNAzymes**	Single-stranded oligo-deoxyribonucleotide molecules.	Catalytic cleavage of target mRNA, inhibiting cancer cell proliferation.	Targeting *BIRC5*, *Bcl-2*, and other anti-apoptotic proteins; inducing cell cycle arrest.	DNAzyme-nanoparticle complexes, cell-penetrating peptides	[81,86]
**tFNA**	Tetrahedral framework nucleic acids modified with microRNA protein.	Modified with microRNA targeting the *BIRC5* gene, expression reduction, and targeted delivery of therapeutic agents.	Delivering siRNAs, miRNAs, and chemotherapeutic agents to cancer cells with high specificity.	Tetrahedral DNA nanostructures, bio-barcode nano platforms	[28,87]

Nucleic acid therapy is an essential principle that enables the development of new genetic disorder treatments by controlling gene expression at the molecular level. This therapeutic method involves the introduction of nucleic acids into cells to correct, enhance, or suppress gene expression, thereby treating diseases, including cancer, genetic disorders, and metabolic diseases [88]. Scientists have created nucleic acid therapeutics featuring antisense oligonucleotides (ASOs), siRNA, and RNA aptamers to deliver targeted genetic sequence interventions for treating genetic disorders [89]. Nucleic acids demonstrate therapeutic properties because they control gene expression patterns using RNA interference to disable target disease genes or manipulate splicing with ASOs [90]. The therapeutic effectiveness of nucleic acids improves following bioconjugation and chemical modification, as these methods achieve better drug stability and targeting. Nucleic acid sensing helps control immune responses, making it essential for tumor immunotherapy and gene therapy applications [91]. The benefits of nucleic acid treatment exceed the pharmaceutical market dominance of small-molecule medicines, as these drugs rely on their target’s drug abilities.

FNAs’ therapeutic applications of FNAs include gene silencing, inhibition of cancer cell proliferation, regulation of gene expression, and promotion of programmed cell death. RNAi is a well-known FNA mechanism that uses siRNAs and miRNAs to target mRNA degradation, reducing oncogene expression and amplifying tumor suppressor genes [92]. ASOs are another FNA strategy that binds mRNA precisely to suppress gene expression. This method was applied to silence the *MET* gene (c-Met), which promotes tumor growth and metastasis. Tetrahedral framework nucleic acids (tFNA) modified with microRNA targeting the *BIRC5* (surviving) gene prove expression reduction, resulting in FNA activation apoptotic markers and cell cycle arrest in cancer cells [28,87]. FNAs can modify the apoptotic process in two ways: by direct interaction with apoptotic proteins or by altering the gene expression patterns that control p53 and supplementary apoptotic regulators [93]. Furthermore, FNAs demonstrate such versatility in programming capabilities that they maintain a central role in future advancements in personalized cancer therapy [70]. They activate apoptosis through various pathways while providing prospects for enhancing drug resistance in cancer cells.

The delivery of functional nucleic acids, including RNA and DNA, is critical for gene therapy and other therapeutic applications. However, these drugs have a short shelf life and poor biological penetration; therefore, scientists must develop sophisticated delivery methods that combine nanoparticles with viral vectors to transport the compounds effectively [68]. The development of both viral and non-viral nanotechnology-based systems has advanced barrier-penetration capabilities in medicine because of nanoparticles that present lower immune responses and improved targeting specificity [94,95,96]. Nanotechnology, bioengineering research, and technological progress have improved the development of harmless and more efficient delivery vehicles for nucleic acids [97,98]. Moreover, recent studies have demonstrated that nanocarriers enhance SFN bioavailability and solubility, improving FNA delivery in cancer treatment [99]. Target cell transfection and biological barrier penetration need effective delivery systems to reach their goal.

## 4. SFN-Based Functional Nucleic Acids for Cancer Therapy

### 4.1. Design and Synthesis of SFN-Based FNAs

The strong nucleic acid attraction of SFN modifies the structural and biological components of nucleic acids. The binding affinity between SFN toward nucleic acids is relatively strong, which reveal intercalation and groove binding mechanisms. The binding (association) constants (Kₐ) = 3.01 × 10^6^ M^−1^ for DNA and = 6.63 × 10^5^ M^−1^ for RNA [100], indicating a higher affinity of SFN for DNA compared to RNA. The development of sulforaphane-based FNAs uses new procedures that capitalize on the distinct features of SFN and its analogs. The chemical modification procedure builds sulfur connections at specific DNA and RNA backbone sites through post synthetic structural changes. The use of sulfinate reagents to modify nucleic acids enhances their strength against enzymatic degradation, making them better applicants for therapeutic applications [101,102].

Quantitative structure-activity relationships (QSAR) modeling and molecular cutting are computational methods that improve the design process of novel SFN analogs to support potential applications in cancer treatment [103]. The detection sensitivity of electrochemical sensors increases through ligand-binding interactions with FNAs. The unique composition of SFN derivatives and FNAs provides precise molecular binding capabilities for the development of therapeutic and diagnostic innovations [104]. Multiple chemical properties and principles govern the design of sulforaphane-based functional nanoparticles to enhance their stability and bioavailability. SFN serves as a potent anticancer compound; however, research groups have encapsulated it into nanoparticles because of its instability and poor solution behavior in biological environments [105]. Newly developed sulforaphane analogs show improved anti-cancer properties through compounds like SF102, which outperforms SFN in halting tumor growth while displaying modifications to achieve better results [106].

### 4.2. Mechanisms of SFN-Based FNAs-Mediated Anti-Cancer Effects

**Figure 5:**
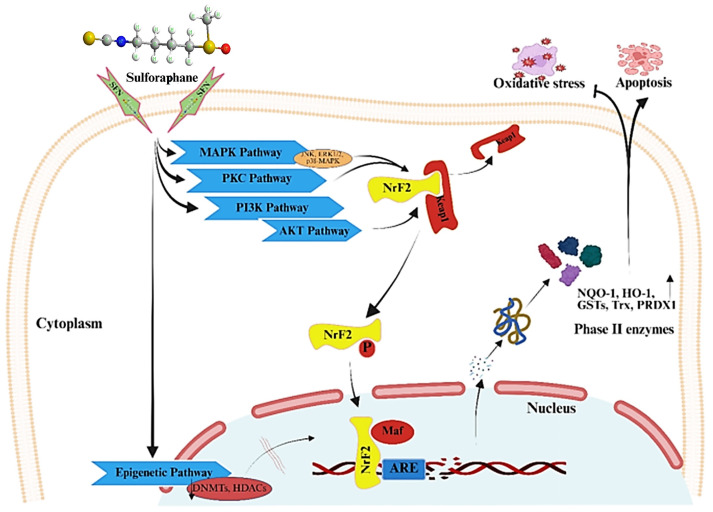
Schematic representation of the Nrf2 signaling pathway activated by sulforaphane (SFN). SFN stimulates MAPK, PKC, and PI3K/AKT pathways, leading to the release and phosphorylation of Nrf2 from Keap1. Phosphorylated Nrf2 translocates to the nucleus, where it binds to Maf and the antioxidant response element (ARE), inducing transcription of Phase II antioxidant and detoxification enzymes (NQO-1, HO-1, GSTs, Trx, PRDX1). The pathway also modulates oxidative stress and apoptosis. SFN additionally affects epigenetic regulators (DNMTs and HDACs). Arrow styles indicate different types of interactions.

The natural isothiocyanate SFN is a major broccoli derivative that exhibits powerful cancer-fighting properties through diverse biological pathways. SFN executes its antioxidant functions by activating Nrf2 signaling (Figure 5), which activates phase II detoxification enzymes and eliminates ROS [107]. The cancer cell targeting efficiency of FNAs improves when using SFN because it functions through several molecular mechanisms that enhance the delivery. The dual mechanism of action of SFN leads to ROS production by inhibiting the phosphatidylinositol-3-kinase (PI3K)/Akt and cyclooxygenase-2 pathways, which triggers various cellular survival pathways and mitochondrial dysfunction, facilitating the apoptosis of cancer cells, Figure 5 [108,109]. Oxygen-derived ROS activate AMPK to drive cellular death processes while triggering cell cycle arrest due to their influence on apoptotic signaling pathways. Breast cancer cells respond to SFN through autophagic mechanisms and mTOR pathway suppression, which jointly induce cell cycle arrest and death [110,111]. SFN sensitizes TRAIL-resistant hepatoma cells to TRAIL-induced apoptosis through its ability to enhance the death receptor DR5 and generate ROS that strengthen apoptotic signaling [112]. Apoptosis induction by SFN occurs in colorectal cancer cells by inhibiting the cyclooxygenase-2/PKB/GSK-3β signaling pathway, demonstrating its cancer cell-specific targeting effects [113]. SFN has various anticancer modalities by increasing *NLRP12* levels, thus regulating the conventional and nonconventional NF-κB signaling pathways to impede cancer cell migration and invasion in lung adenocarcinoma cells [60,114].

The cancer cell-killing properties of SFN remain strong, yet the substance exhibits low toxicity in normal cells. The viability of cancer cells decreased by approximately 75% in HepG2 cells treated with 32 µM SFN for 96 h. Meanwhile, treating human hepatocytes with 50 µM SFN over 48 h periods caused no detrimental effects [42,115]. When applied to lung cancer cell lines, SFN functionally decreased HDAC activity, triggering increased histones H3 and H4 acetylation. It also has numerous anti-cancer effects, including the activation of apoptosis mechanisms and cell cycle arrest in the S phase, followed by cell accumulation in the G0/G1 and G2/M phases. Treatment with SFN led to a pronounced reduction in the proliferation of lung cancer cells during xenograft research on mice [116]. A previous study used BALB/c nude mice that received SFN supplementation during the pre-inoculation period and following cell inoculation by administering 50 mg SFN/kg through daily intraperitoneal injections for 5 and 3 weeks. These in vivo research methods assessed the effects of SFN on initial tumor formation and further development in mammary tissues using NanoString gene testing. Scientists have discovered that SFN reduces both the proliferation potential and formation of mammosphere cells in TNBC CSCs [117]. The suppression of histone deacetylase enzyme activity and activation of the ERK signaling pathway led to significant antiproliferative effects of SFN on schwannoma cells in culture conditions. In vivo tests have shown that SFN efficiently suppresses schwannoma tumors, signifying its potential for pharmaceutical use [118].

## 5. Key Targets Regulation of SFN-Based FNAs as a Therapeutic Strategy for Carcinogenesis

### 5.1. Transcriptional Targets

#### 5.1.1. Nrf2 Pathway: The Master Regulator of the Body’s Antioxidant Pathway

Nrf2 is a transcription factor (TF) that is a member of the Cap ‘N’ Collar (CNC) subfamily of basic leucine zipper (bZip) proteins, which plays an important role in the cellular stress response to oxidative insults of metabolic, chemical, or pathological origin [119]. Nrf2 promotes the survival of normal cells, cancer cells, and cancer stem cells (CSCs) as a result of amino acid metabolism activated in normal and tumor cells in response to stress under pathological conditions through regulation of the expression of enzymes involved in redox homeostasis, cellular metabolism, and xenobiotic detoxification [120]. Nrf2 is a master transcription factor that senses oxidative stress and induces the transcription of genes responsible for detoxification and antioxidant responses, such as *glutathione-S-transferase*, *heme oxygenase-1*, and NAD(P)H: *quinone oxidoreductase 1*, which are critical for preventing oxidative damage [121]. Kelch-like ECH-associated protein 1 (*KEAP1*) is a substrate-specific adaptor protein of the Cullin 3 (CUL3)-based E3 ubiquitin ligase complex that serves as a primary sensor of oxidative and electrophilic stress in cells, the main regulator and canonical interactor of NRF2, and controls NRF2 levels under physiological conditions. However, under conditions of oxidative stress or in the presence of electrophilic compounds, the redox-sensitive cysteine residues (for example, Cys151, Cys273, and Cys288) in *KEAP1* are modified, leading to Nrf2 dissociation and eventual nuclear translocation. Nrf2 heterodimerizes with small musculoaponeurotic fibrosarcomas (sMaf) and other transcription factors, including SP-1 or c-JUN, binds to specific sequences called antioxidant responsive elements (AREs) in the promoter region of various target genes; and induces the transactivation of important antioxidant genes, including *NQO1*, *HMOX1*, *GSR* (Glutathione-Disulfide reductase), *PRDX1* (peroxiredoxin 1), and *SLC7A11* [122,123] (Figure 5).

SFN, which induces Nrf2 activation in cancer cells, is known for its anticancer activity. By improving antioxidant enzymes and supporting cancer cell survival during oxidative stress, activation of Nrf2, in particular, promotes cell proliferation in certain situations, such as HCT116 colon cancer cells with wild-type p53 expression [58,124]. In addition, SFN-induced Nrf2 activation is linked to resistance to different therapies, presenting a double-edged sword for cancer cell survival and therapy resistance [125]. Nevertheless, Nrf2 plays an ambiguous role; apart from inhibiting carcinogenesis, Nrf2 hyperactivation in malignant tumors enhances cell turnover, invasiveness, and treatment resistance, leading to adverse clinical outcomes [126]. On the one hand, Nrf2 protects healthy cells from oxidative damage, but when overexpressed in cancer cells, it can promote therapy resistance. Consequently, it represents a target for new therapeutic strategies in oncology [127].

#### 5.1.2. MAPK Pathway

The MAPK pathway is a critical signaling cascade that regulates many cellular processes and physiological phenomena, such as proliferation, differentiation, and apoptosis. This pathway relays signals from receptor tyrosine kinases (RTKs). It incorporates feedback loops that influence cellular responses to external factors, such as proliferation, differentiation, apoptosis/survival, innate immunity, and inflammation [128]. SFN induced the MAPK pathway, a well-known signaling pathway involved in cancer signaling and apoptosis. Studies have shown that it acts by increasing the activation of p38 MAPK, which upregulates the expression of ARE-dependent enzymes while simultaneously downregulating cyclooxygenase-2 (COX-2) in various types of cancer cells, including bladder and breast cancer cells. SFN is also known to exert effects on the MEK/ERK pathway, which might be involved in the anti-proliferative effects of SFN on glioma [129]. At the molecular level, SFN activates the phosphorylation of ERK1/2 and p38 MAPK, which cause apoptosis in different cancer cell lines, such as colon cancer and non-small cell lungs cancer cell lines. The activation of ERK1/2 plays an important role in procaspase 9 cleavage, enabling apoptosis through the degradation of pro-survival proteins such as Bim [114], Figure 5.

In triple-negative breast cancer (TNBC), SFN decreases cell proliferation, tumor growth, and metastasis, making it a promising therapeutic agent. Moreover, SFN inhibits the CDC2/cyclin B1 complex, resulting in a mitotic delay and inhibition of cell proliferation in breast cancer cells [52]. In addition, SFN plays a crucial role in promoting cell survival inhibition and suppressing proliferation in colorectal cancer cells, primarily by downregulating the cyclooxygenase-2 (COX-2)/PI3K/Akt signaling pathway [113]. In addition, SFN promotes the expression of UDP glucuronosyltransferase 1A (*UGT1A*) through the ERK/Nrf2 signaling pathways, detoxifying carcinogens and inhibiting cancer cell proliferation [130]. These findings collectively indicate the requirement for SFN as an important agent in cancer therapy with multiple targets. Also multifaceted mechanisms by which SFN drives cancer prevention and treatment by targeting cancer cell proliferation via the MAPK and MAPK-related signaling cascades.

#### 5.1.3. Nrf2-ARE (Antioxidant Response Element) Pathway

Antioxidants protect cells by activating the Nrf2-ARE pathway, an important cellular defense system that is highly regulated by the transcription factor Nrf2. This pathway is activated when Nrf2 dissociates from its repressor *Keap1*, translocate to the nucleus, and associates with AREs, promoting the expression of many antioxidant and cytoprotective genes such as *NQO1*, *HMOX1*, and glutamate–cysteine ligase modifier (*GCLM)* subunit [131]. Such cellular redox homeostasis is significantly regulated by the Nrf2/ARE pathway, which is important for the upregulation of cytoprotective genes and enzymes in harmful environments, rendering a cytoprotective response to alleviate inflammation, oxidative damage, and tumorigenesis [132]. SFN is an Nrf2-ARE pathway agonist that enhances antioxidant responses. SFN modifies the Keap1 protein, leading to the destabilization of Nrf2 and subsequent translocation of Nrf2 to the nucleus, where it binds to AREs and induces the expression of cytoprotective genes, such as detoxification and antioxidant genes [53], Figure 5. Furthermore, SFN reverses epigenetic modifications, resulting in the suppression of tumor-suppressor genes [133] thus increasing the expression of downstream target genes involved in cell cycle control, apoptosis, and inhibition of angiogenesis.

Importantly, although Nrf2 activation by SFN may inhibit the development of chemically induced cancers, it may be a pro-tumorigenic factor for the promotion of existing tumors, underscoring the bi-directionality of Nrf2 activity in cancer biology [134]. The Nrf2-ARE pathway activated by SFN in cancer cells promotes the transcription of ARE-dependent genes that play significant roles in cellular defense against oxidative stress. Particularly, main downstream targets of SFN are *HMOX1* and *HSPA1A*, which are significantly upregulated upon SFN treatment and could qualify as markers of therapeutic efficacy in HNSCC [135]. Additionally, KEAP1-Nrf2 plays a core role in the cellular response to oxidative stress, which is involved in various processes related to cancer and could be considered a prognostic and therapeutic target [136]. The Nrf2-ARE pathway is a potential target for cancer prevention and therapeutic strategies that utilize SFN.

#### 5.1.4. NF-κB Pathway: SFN Can Inhibit NF-κB, a Transcription Factor Involved in Inflammation and Cancer Progression

The NF-κB pathway is a ubiquitous signaling pathway important for immune response, inflammation, and cell survival. Containing five members (RelA [p65], RelB, c-Rel, p50, and p52), NF-κB serves as a dominant regulator of inflammatory and immune responses by regulating cytokine production and immune cell differentiation [137]. Naturally, they are inhibited by the IκB family of proteins or a self-inhibitory domain (NF-κB1 and NF-κB2). Translocation of NF-κB from the cytosol to the nucleus and activation of target genes to pro-tumorigenic effects during chronic inflammation by oxidative stress is mediated by the separation of IκB molecules or cleavage of the inhibitory domains of NF-κB1 and NF-κB2 as an important requirement [138]. The underlying mechanism of action of SFN in cancer cell proliferation and survival is mainly mediated by the inhibition of NF-κB activation. In specific, SFN downregulates the expression and phosphorylation of NF-κB and matrix metalloproteinase-9 (*MMP-9*), resulting in reduced cell proliferation and enhanced apoptosis in the breast cancer cell lines MDA-MB-231 and MCF-7 [48].

SFN inhibits the evolutionary transcription factor NF-κB, a key regulator of inflammation and cancer. SFN is a negative regulator of NF-κB activation that downregulates the production of pro-inflammatory cytokines, such as IL-1β and IL-8, associated with numerous inflammatory diseases and cancer [139]. In vivo, SFN reduced tumor volume and colitis-associated carcinogenesis, suggesting that it could restructure the inflammatory microenvironment and enhance immune responses [140]. As a chemo preventive agent, SFN proves benefits not only related to the inhibition of tumor growth by the modulation of various cellular targets, such as NF-κB, but also through the sensitization of cancer cells to chemotherapy response [141]. These mechanisms provide the basis for SFN’s potential in countering inflammation and cancer progression by inhibiting the NF-κB pathway. Together, these mechanisms emphasize the therapeutic potential of SFN in cancer therapy by targeting the important survival pathways in cancer cells.

#### 5.1.5. Activator Protein-1 (AP-1): SFN Can Inhibit AP-1, a Transcription Factor Involved in Cell Proliferation and Survival in Cancer Cells

AP-1 is a transcription factor that represents a group of heterodimeric complexes and is responsible for facilitating a wide variety of biological processes, including cell proliferation, cell differentiation, apoptosis, survival, cell transformation, and migration [142]. AP-1 is a group of JUN and FOS proteins family formed by several subunits, that modulate gene expression by binding to specific DNA sequences through a preserved basic-zipper domain [143]. The AP-1 complex has been linked to the development and progression of inflammatory diseases, such as asthma, cancer, rheumatoid arthritis, and psoriasis. AP-1 targets genes involved in cell growth, apoptosis, and invasion; therefore, it is a potential target for cancer therapy. Additionally, the activity of AP-1 is influenced by many stimuli, such as growth factors and cytokines, which activate the MAPK pathway to enhance the expression of numerous oncogenic genes [144].

Research has shown that SFN inhibits transcription factor AP-1, an important mediator of many oncogenic signaling pathways. SFN inhibits IL-1β-induced IL-6 expression through ROS production and MAPK/AP-1 signaling in colorectal cancer [47]. In addition, SFN downregulates nicotine-mediated *MMP-9* expression in gastric cancer cells by ROS-mediated suppression of the AP-1 and NF-κB signaling cascades [46]. Overall, these findings suggest that SFN acts as a potent modulator of the AP-1 pathway, clarifying the mechanisms underlying SFN-induced apoptosis and the importance of SFN’s anticancer effects, and suggesting that it is a promising pharmacological agent for tumor therapy.

### 5.2. Post-Transcriptional Targets

#### 5.2.1. MicroRNAs (miRNAs): SFN Can Modulate miRNA Expression, Thus Altering Gene Expression and Cancer Cell Behavior

miRNAs are 18–25-nucleotide endogenous, non-coding, single-strand RNAs. MiRNAs are generally generated in intergenic regions, introns, exons, or target genes’ 3′-untranslated regions (3′-UTR) [145]. miRNA genes are typically transcribed by RNA polymerase II, and during their maturation process, they contain multiple RNA forms. The full-length precursor of miRNA, pri-miRNA, is formed first, approximately 300–1000 nts long. Unlike mature miRNAs, pri-miRNA transcripts are analogous to those of classic protein-coding genes modified with a non-canonical poly-A tail at the 3 ′ end and a 5-end nucleotide cap [146]. miRNAs repress target genes by inhibiting mRNA translation or promoting mRNA degradation. Communally, miRNA joins with Argonaute (Ago) protein to generate a complicated and subsequently transported into the nucleus to couplings the sequence using total or incomplete complementarity. With this active complex, multiple agents such as RNA polymerase II, transcriptional factors, or histone-modifying enzymes are recruited to the regulatory regions, leading to transcriptional activation or epigenetic modification of target genes [147].

In particular, SFN treatment is emerging as a promising therapeutic approach for cancer management through the modulation of miRNA expression. Research shows that SFN can significantly affect different miRNAs related to cancer progression, including *miR-15b-5p* and *miR-9-3*, which may promote apoptosis and inhibit the proliferation of cancer cells [148]. SFN treatment of breast ductal carcinoma in situ stem-like subpopulations: Upregulated exosomal *miR-140* and downregulation of *miR-21* and *miR-29*, SFN also inhibited colony formation and ALDH1 expression [149]. SFN was found to upregulate *let-7f-5p* and downregulate *miR-29b-3p*, both of which are involved in suppressing oncogenes such as *HMGA2*, *CDC25A*, and *MYC* in colorectal cancer [150], Table 3. In colorectal cancer, SFN downregulates oncogenic *miR-21* and human telomerase reverse transcriptase (*hTERT*), leading to decreased cell viability and induced apoptosis [151].

#### 5.2.2. mRNA: SFN Can Bind to mRNA and Inhibit Translation to Decrease the Expression of Oncogenic Proteins

mRNA is a vital macromolecule in biology that acts as a master for synthesizing proteins and is involved in main cellular mechanisms such as gene expression and regulation. The recent progresses in mRNA have demonstrated to be significantly helpful in treating several diseases, including cancer and regenerative medicine [152,153]. SFN bind mRNA and inhibit its translation, leading to decreased oncogenic protein expression. Studies have shown that SFN interacts with multiple cellular pathways, including pro-apoptotic, cell cycle inhibition, and phase II detoxification enzyme upregulation mediated by Nrf2 [109], Table 3. In particular, SFN has been shown to suppress pancreatic cancer development by inhibiting long noncoding RNA *H19* and its target *APOBEC3G* [154].

SFN promotes the upregulation of *miR-199a-5p*, which downregulates the mRNA levels of *Sirt1* and *CD44ICD*, resulting in reduced proliferation and invasion of skin squamous cell carcinoma cells [155]. The National Cancer Institute (NCI) has indicated that the growth-inhibitory effect of SFN induces apoptosis in numerous cancer types, such as TNBC, is selective for the cell type, and depends on multiple pathways [52]. Together, these results emphasize the multifactorial nature of SFN as a cancer therapeutic, including its ability to reduce the translation of oncogenic mRNAs.

### 5.3. FNAs Targets

#### 5.3.1. Aptamers: SFN-Based Aptamers Can Bind to Target Proteins on the Cancer Cell Surface, Transforming Their Signaling Pathways and Behavior

Aptamers are short single-stranded DNA or RNA oligonucleotides that specifically recognize and bind to target molecules through the formation of characteristic secondary and tertiary structures resulting from their folding [156]. They have high specificity and affinity, can target various well-known cancer biomarkers, and can be engineered to bind specifically to receptors overexpressed on tumor cells, enhancing the targeted delivery of therapeutic agents such as SFN [60]. This selectivity enables early diagnosis, which is essential for enhancing patient diagnosis and therapeutic efficacy. In addition, aptamers can be applied to targeted drug delivery systems, reducing side effects and increasing therapeutic effects by directing drugs to cancer cells [157]. Aptamer-mediated targeting with SFN derivatives can be achieved by developing nucleic acid ligands that bind to a specific protein and deliver SFN.

Thus, aptamers similar to SFN could be valuable for targeting specific cancer cell surface proteins that affect cell signaling and characteristics. For example, *KRAS*-binding aptamers can block KRAS interaction with Raf-1 kinase, a key member of a pathway that regulates cell growth, thereby inhibiting cancer growth [158]. SFN-based aptamer-induced apoptosis in cancer cells likely occurs through several interconnected mechanisms, primarily via ROS generation and modulation of key signaling pathways. Disruption of the PI3K-PKB/Akt pathway is a key route that induces mitochondrial dysfunction and caspase-independent apoptosis [109], Figure 5. In general, sulforaphane-based aptamers in targeted cancer diagnosis and therapy also improve the efficiency of diagnosis and decrease the side effects of conventional therapeutics.

#### 5.3.2. Small Interfering RNA (siRNA): SFN-Based siRNAs Can Silence Specific Oncogenes, Decreasing Cancer Cell Proliferation and Survival

SFN-based siRNA therapeutics have demonstrated therapeutic efficacy in multiple cancer cell types through various mechanistic pathways. SFN, when coupled with siRNA technology, may target specific genes that promote cancer progression in a manner that uses the gene-silencing power of siRNA to increase treatment efficacy [159]. For example, lipid nanoparticles are an advanced delivery system that can transport siRNA into cancer cells to avoid off-target effects while increasing the specific targeting ability [160]. Moreover, the observation that siRNA-mediated voicing of stathmin1 (*STMN1*) in prostate cancer cells is associated with diminished proliferation rates and increased apoptotic signaling highlights the potential of targeted gene silencing for treatment applications [39].

Numerous studies have demonstrated that SFN-based siRNAs silence target genes in a highly specific manner to induce significant cancer cell death and inhibit cell growth and proliferation. SFN may stimulate cell cycle arrest and apoptosis in breast cancer cells by altering the expression of critical regulatory proteins, including *CDK5R1* and *Bcl-2*, ultimately resulting in inhibited cell growth and elevated apoptosis markers [52]. For instance, in pancreatic cancer, SFN exploits the oncogenic long noncoding RNA *H19* as a therapeutic target to repress its expression and that of its target, the antiviral *APOBEC3G*, leading to the attenuation of tumor growth and apoptosis [154], Table 3. Together, these data demonstrate that the selectivity of SFN-based siRNAs can effectively interfere with pathways essential for cancer cell survival/proliferation, ultimately leading to their death.

### 5.4. Epigenetic Targets

Histone modifications and DNA methylation are the principal molecular mechanisms that constitute the epigenetic process regulated by histone deacetylases (HDACs) and methyltransferases (DNMTs), respectively. Specifically, SFN has been shown to affect epigenetic mechanisms, modulating gene expression and ultimately cellular function, Figure 5. Enzymes that mediate the epigenetic signature of cancer cells are targeted in cancer prevention and therapeutic studies.

DNA methylation regulates gene activity and can regulate entire DNA domains. It is a chemical and biological modification at the 5′position of the cytosine residue. This modification predominantly occurs at cytosine residues found within GC dinucleotide-rich areas that aggregate to form CpG that extends the 5′end area of many genes.

#### 5.4.1. DNMTs: SFN Inhibits DNMTs, Decreases DNA Methylation, and Reactivates Tumor Suppressor Genes

DNMTs are a family of enzymes that catalyze methylation transfer to DNA. Inhibition of these enzymes promotes apoptosis and halts the cell cycle. SFN plays an active role in downregulating the functions of DNMTs (particularly *DNMT1* and *DNMT3B*) and is therefore protective against cancer [161]. In breast cancer cells, SFN-induced G1 arrest, nitro-oxidative stress, and genotoxicity were correlated with global DNA hypomethylation, a decrease in DNA methyltransferase 1 and 3B levels, reduced pools of N^6^-methyladenosine RNA methylation, and alterations in the microRNA profile (upregulation of 60 microRNAs and downregulation of thirty-two microRNAs) [162]. In prostate cancer cells, SFN markedly reduced the expression of DNMTs and reactivated methylation-silenced cyclin D2 (*CCND2*) [163]. These results reveal the epigenetic modulatory ability of SFN on cyclin D2 expression and provide novel insights into the mechanisms by which SFN may regulate gene expression as a chemopreventive agent in prostate cancer. SFN increased the mRNA and protein expression of Nrf2 and its target gene *NQO1* by dampening the protein levels of *DNMT1* and *DNMT3a* [164], Table 3.

SFN, a compound found in cruciferous vegetables, reduces the protein expression of HDAC1, 2, 4, and 6 in human malignant melanoma cells. SFN also downregulated total HDAC activity and the protein levels of CBP, CBP/p300, and PCAF. Moreover, SFN treatment markedly decreased the protein expression levels of acH3K9, acH3K14, acH3K27, acH3K8, and acH3K12. Notably, H3K36 and H3K79 methylation was reduced during SFN treatment, resulting in decreased HMT activity at H3K9, H3K36, and H3K79 [165]. SFN inhibited the expression of DNMT1 in colon cancer cells and decreases Nrf2 promoter region methylation, thereby enhancing Nrf2 protein expression [166]. SFN-regulated expression of putative tumor suppressor genes, phosphatase and tensin homolog (*PTEN*), and retinoic acid receptor beta 2 (*RARbeta2*) is also due to hypomethylation of tumor suppressor gene promoters through DNMT inhibition [167]. SFN demethylates the CpG sites of the tumor suppressor *miR-9-3* promoter and reactivates *miR-9-3* expression by suppressing DNMT activity, *DNMT3a* protein expression, and histone deacetylase 1, 3, and 6 in the human lung cancer cell line, A549 [168], Table 3. This can reactivate silenced genes, restore cell functions, and help prevent or treat cancer. DNMT inhibition by sulforaphane may prevent hypermethylation of tumor suppressor genes and inhibit cancer development.

#### 5.4.2. Histone Modification

Histone modifications are remarkable regulators of chromatin dynamics that govern gene regulation, DNA repair mechanisms, cell proliferation, and apoptosis. Deregulated genes in these processes can lead to the unintended activation of oncogenes or inactivation of tumor suppressor genes in cancer [169].

#### 5.4.3. A Histone Deacetylases (HDACs): SFN Can Inhibit HDACs, Increasing Histone Acetylation and Activating Tumor Suppressor Genes

HDAC caused by HDACs represents an important epigenetic alteration in malignant cells. HDACs are enzymes that facilitate the classical removal of acetyl groups from histones and other proteins, leading to chromatin reorganization and alterations in the gene expression profile [170]. SFN is a cellular epigenetic inhibitor that modulates HDAC enzymes that deacetylate histones and turn off DNA, resulting in tighter DNA packaging and gene transcription repression [171]. SFN promotes HDAC by inhibiting HDACs, thereby relaxing the chromatin structure and promoting the transcription of genes. This can activate tumor suppressor and other protective genes. In cancer cells, SFN inhibits HDACs. Inhibiting HDACs is essential for cancer prevention as it promotes several mechanisms, such as apoptosis and cell cycle arrest [42]. SFN inhibited *HDACs5* transcription by antagonizing USF1 (upstream transcription factor 1) activity in breast cancer cells and blocking the HDAC5-lysine-specific demethylase 1 axis to suppress breast cancer growth. An increase in HDAC also occurs at the promoters of silenced tumor suppressor genes, which is accompanied by increased expression of the genes [172], Table 3.

Prostate cancer cells frequently undergo silencing of the gene encoding the tumor suppressor p21 (*CDKN1A*). Sulforaphane treatment increases promoter acetylation and promotes p21 expression. This effect was also noted in the p53 null prostate cancer cell line PC3, indicating epigenetic reactivation [173]. In another study, mice were administered a single 10 µmol oral dose of SFN by gavage, revealing significant inhibition of HDAC activity in the colonic mucosa after 6 h and an immunoblot demonstrating a parallel increase in acetylated histones H3 and H4, which reverted to control levels at 48 h. Increased levels of acetylated histones and p21(*WAF1*) in the ileum, colon, prostate, and peripheral blood mononuclear cells were observed after longer-term treatment (10-week study) with SFN in the diet (443 mg/kg) [174]. SFN-mediated tumor suppression in Apc-minus mice was linked to the inhibition of HDAC activity and increased histone H3 and H4 acetylation within the promoter regions of *CDKN1A* and *Bax* genes [175]. In cancer, increased histone acetylation can activate oncogenes and inhibit proteins that prevent cell division, induce apoptosis, or cause DNA damage, thus suppressing tumor growth and being involved in treatment. SFN may be a potential therapeutic agent, as it can induce apoptosis in cancer cells by modulating histone acetylation.

**Table 3:** Transcriptional, Post-Transcriptional and Epigenetic Targets of SFN-Based FNAs.

Target Pathway/Type	Description	Key Molecules	Cancer Types	References
**Nrf2 Pathway**	SFN activates Nrf2, leading to the upregulation of antioxidant and detoxification enzymes.	Nrf2, ARE, *HMOX1*, *NQO1*, *GSR*, *PRDX1*	Breast, Bladder, Liver, Lung, Colon	[53,122]
		*HMOX1*, *NQO1*, *GSR*,	Bladder cancer cells	[176]
**MAPK Pathway**	SFN activates MAPKs, leading to apoptosis and cell cycle arrest.	ERK, JNK, p38, COX-2	Pancreatic, Breast, Colon, Lung	[108,114]
		P53, P27, *Bax*, *Bcl-2*, *CCND1*, Her2	Osteosarcoma	[44]
**Nrf2-ARE Pathway**	SFN induces Nrf2 activation, promoting the transcription of ARE-dependent genes.	Nrf2, ARE, *NQO1*, *HMOX1*, *HSPA1A*,	HNSCC and Various cancers	[43,53]
		Caspase-3, *Bax*, ROS, *Bcl-2*, STAT3	Glioblastoma (Malignant Brain Tumor)	[41]
**NF-κB Pathway**	SFN inhibits NF-κB, reducing inflammation and cancer progression.	NF-κB, IL-1β, IL-8, *MMP-9*	Pancreatic, Breast	[46,139]
		NF-κB, *MMP-9*	Breast cancer	[48]
**AP-1 Pathway**	SFN inhibits AP-1, affecting cell proliferation and survival.	AP-1, IL-1β-induced IL-6, *MMP-9*	Colorectal, Gastric	[46,47]
		HIF-1α, VEGF, AP-1, *MMP-9*, *COL3A1*, *COL5A1*,	Gastric cancer	[57]
**miRNAs**	SFN modulates miRNA expression, altering gene expression and cancer cell behavior.	*miR-15b-5p*, *miR-9-3*, *miR-140*	Breast, Colorectal, Lung	[148,149]
		*miR-19*, miR200c, GSK3β, Wnt/β-catenin	Lung cancer	[177]
**mRNA**	SFN binds to mRNA, inhibiting translation and reducing oncogenic protein expression.	*Sirt1*, *CD44ICD*, *hTERT*	Skin, Colorectal, Triple-negative Breast Cancer	[151,178]
		*SLC7A11*, *GSH*	Small-cell lung cancer	[179]
**DNA Methylation**	SFN inhibits DNMTs, leading to hypomethylation and reactivation of tumor suppressor genes. Inhibition of *DNMT1* expression	*DNMT1*, *DNMT3B*	Reactivation of silenced genes, apoptosis	[42,163]
		Restoration of P21, *PTEN*, and *RARbeta2* Cell growth arrest and apoptosis	Human breast cancer cells	[167]
**Histone Modification**	SFN inhibits HDACs, increasing histone acetylation and activating tumor suppressor genes. Inhibition of class I and II HDACs activity	HDAC1, HDAC2, HDAC4, HDAC5	Increased gene expression, cell cycle arrest, apoptosis	[116]
		Reactivation of *CDKN1A* and *Bax* Cell cycle arrest and apoptosis	Prostate cancer cells Lung cancer cells	[27,173]

## 6. Challenges and Future Directions

Although SFN has shown promising anticancer effects and the potential to synergize with FNAs, several challenges must be overcome to translate these discoveries into clinical applications. A significant limitation of SFN application is its physiochemical instability and rapid metabolism, which restricts its therapeutic efficacy. To improve the stability of SFN and its targeted delivery to the tumor site, SFN can be incorporated into nanoparticles and chemically modified. Next-generation delivery systems, such as lipid nanoparticles and viral vectors, have been developed to enhance cellular uptake and reduce degradation. Furthermore, the variability in metabolism between individuals, which is modified by the gut microbiota and genetic polymorphisms, represents a significant barrier to obtaining consistent therapeutic results.

Another challenge is the design and optimization of SFN-based FNAs. However, the in vivo stability and delivery efficiency of aptamers, siRNAs, and miRNAs are still less than ideal. High-throughput screening and computational modeling can aid in the engineering of FNAs with improved specificity. Target specificity and off-target interactions can be further improved using chemical modifications and locked nucleic acids (LNAs). Indeed, the efficient delivery of FNAs to cancer cells remains a crucial challenge because of the lack of cellular uptake and rapid clearance from the bloodstream. Lipid nanoparticles, polymeric nanoparticles, exosomes, and other nanotechnology-based delivery systems have been explored to improve delivery efficiency. Such systems can enhance the intracellular internalization and therapeutic potency of SFN-based FNAs, mainly targeting cancer cells. Additionally, the diverse signaling pathways modulated by SFN necessitate a better delineation of the mechanisms through which SFN exerts its activity, which is particularly important considering the heterogeneity of cancer and resistance. However, further studies are needed to define the molecular targets of SFN and design combinations that augment its activity.

It should also be emphasized that large-scale clinical trials are needed to confirm the safety and efficacy of SFN-based therapies in various types of cancer and in different patient populations. Finally, to fully realize its anticancer potential, the potential synergistic effects of SFN with other chemopreventive agents and therapeutic modalities remain to be investigated. Further development of innovative delivery carriers, such as viral-like particles and cell-penetrating peptides, is needed to improve the delivery efficacy and specificity of SFN-based FNAs. High-throughput screening and genomic profiling should be used to elucidate patient-specific targets and optimize SFN-based FNA therapies for individual patients. Creating tailored treatment plans according to the unique genetic and epigenetic profiles of tumors improves therapeutic results and decreases side effects. Lastly, the inclusion of artificial intelligence and computational modeling may accelerate the development of SFN analogs with improved anticancer efficacy and reduced toxicity.

## 7. Conclusions

SFN is a powerful natural compound with anticancer activity that provides a multi-faceted approach to cancer therapy and prevention. As a promising therapeutic agent, SFN effectively modulates potent intracellular signaling pathways to induce apoptosis and strengthen detoxification mechanisms. It serves as a carrier of FNAs (siRNAs, miRNAs, and aptamers). It holds powerful potential for targeted cancer treatment, improving therapeutic efficacy, and decreasing off-target effects in conventional cancer treatments. Indeed, further developments in SFN bioavailability, delivery, and clinical translation are required to fulfill its therapeutic promise. The epigenetic mechanisms of SFN should be further studied to develop novel cancer treatments, and the delivery system should be optimized and evaluated in a clinical setting. By combining the best natural active ingredients with cutting-edge nucleic acid-based technologies, SFN-based FNAs can transform cancer treatment from a cocktail of toxic drugs to a safer, more targeted, and more effective treatment. This review highlights the continued need for interdisciplinary research to realize the full potential of SFN-based FNAs as cancer therapeutics.

## Data Availability

The original contributions presented in this study are included in the article. Further inquiries can be directed at the corresponding authors.

## Abbreviations

The following abbreviations are used in this manuscript:

**Abbreviation**	**Full Term**
AGS	Human gastric adenocarcinoma cell line
AMPK	AMP-activated protein kinase
AP-1	Activator protein-1
APOBEC3G	Apolipoprotein B mRNA-editing enzyme catalytic subunit 3G
ARE	Antioxidant response element
ASO	Antisense oligonucleotide
Bax	Bcl-2-associated X protein
Bcl-2	B-cell lymphoma 2
Bim	Bcl-2-like protein 11
bZip	Basic leucine zipper
CBP	CREB-binding protein
CD44	Cluster of differentiation 44
CD44ICD	CD44 intracellular domain
CDC2	Cell division cycle 2 (CDK1)
CDC25A	Cell division cycle 25A
CDK	Cyclin-dependent kinase
CDK1	Cyclin-dependent kinase 1
CDK5R1	Cyclin-dependent kinase 5 regulatory subunit 1
CNC	Cap ‘N’ Collar (transcription factor subfamily)
COL3A1	Collagen type III alpha 1 chain
COL5A1	Collagen type V alpha 1 chain
COX-2	Cyclooxygenase-2
CSC	Cancer stem cell
CUL3	Cullin 3
CYP	Cytochrome P450
DMSO	Dimethyl sulfoxide
DNA	Deoxyribonucleic acid
DNMT	DNA methyltransferase
DR5	Death receptor 5
EGF	Epidermal growth factor
EGFR	Epidermal growth factor receptor
EMT	Epithelial–mesenchymal transition
ERK	Extracellular signal-regulated kinase
ESP	Epithiospecifier protein
FGF-2	Fibroblast growth factor 2
FNA	Functional nucleic acid
GLOBOCAN	Global Cancer Observatory database
GRN	Glucoraphanin
GSH	Glutathione
GSK-3β	Glycogen synthase kinase 3 beta
GSR	Glutathione-disulfide reductase
GST	Glutathione S-transferase
HCT116	Human colorectal carcinoma cell line
HDAC	Histone deacetylase
HepG2	Human hepatocellular carcinoma cell line
Her2	Human epidermal growth factor receptor 2
HIF-1α	Hypoxia-inducible factor-1 alpha
HMGA2	High mobility group AT-hook 2
HMOX1 (HO-1)	Heme oxygenase-1
HMT	Histone methyltransferase
HNSCC	Head and neck squamous cell carcinoma
HPLC	High-performance liquid chromatography
HSPA1A	Heat shock protein family A member 1A
hTERT	Human telomerase reverse transcriptase
IL-1β	Interleukin-1 beta
IL-6	Interleukin-6
IL-8	Interleukin-8
ITC	Isothiocyanate
JNK	c-Jun N-terminal kinase
KEAP1	Kelch-like ECH-associated protein 1
KRAS	Kirsten rat sarcoma viral oncogene homolog
LNA	Locked nucleic acid
lncRNA	Long non-coding RNA
MAPK	Mitogen-activated protein kinase
MCF-7	Human breast cancer cell line
MDA-MB-231	Human triple-negative breast cancer cell line
MEK	Mitogen-activated protein kinase
miRNA	MicroRNA
MMP	Matrix metalloproteinase
mRNA	Messenger RNA
mTOR	Mammalian target of rapamycin
NAT	N-acetyltransferase
NCI	National Cancer Institute
NF-κB	Nuclear factor kappa B
NLRP12	NLR family pyrin domain containing 12
NOD	Non-obese diabetic
NQO1	NAD(P)H quinone oxidoreductase 1
Nrf2 (NRF2)	Nuclear factor erythroid 2-related factor 2
NSG	NOD scid gamma (mouse model)
p21 (WAF1)	Cyclin-dependent kinase inhibitor 1A
p38 MAPK	p38 mitogen-activated protein kinase
p53	Tumor protein 53
PCAF	p300/CBP-associated factor
PI3K	Phosphatidylinositol-3-kinase
PKB (Akt)	Protein kinase B
PRDX1	Peroxiredoxin 1
PTEN	Phosphatase and tensin homolog
QSAR	Quantitative structure–activity relationship
RARβ2	Retinoic acid receptor beta 2
RISC	RNA-induced silencing complex
RNA	Ribonucleic acid
RNAi	RNA interference
ROS	Reactive oxygen species
RTK	Receptor tyrosine kinase
SELEX	Systematic Evolution of Ligands by Exponential Enrichment
SFN	Sulforaphane
siRNA	Small interfering RNA
SLC7A11	Solute carrier family 7-member 11
sMaf	Small musculoaponeurotic fibrosarcoma proteins
SP-1	Specificity protein 1
STAT3	Signal transducer and activator of transcription 3
SULT	Sulfotransferase
tFNA	Tetrahedral framework nucleic acid
TNBC	Triple-negative breast cancer
TRAIL	Tumor necrosis factor-related apoptosis-inducing ligand
UDP	Uridine diphosphate
UGT1A	UDP-glucuronosyltransferase 1A
USF1	Upstream transcription factor 1
UTR	Untranslated region
VEGF	Vascular endothelial growth factor
YAP1	Yes-associated protein 1
